# Supplementary material for: Error rate reduction of single-qubit gates via noise-aware decomposition into native gates
Source: Sci Rep. 2022 Apr 16;12:6379. doi: 10.1038/s41598-022-10339-0 (PMC9013363; doi:10.1038/s41598-022-10339-0)
Supplement: Supplementary file 1 — Supplementary Information. [file 41598_2022_10339_MOESM1_ESM.pdf]

Supplementary Information: Error Rate Reduction of Single-Qubit Gates via Noise-Aware Decomposition into Native Gates

Thomas J. Maldonado,[1,2,*] Johannes Flick,[3] Stefan Krastanov,[4,5] and Alexey Galda[6,7,8]

[1]Department of Electrical and Computer Engineering, Princeton University, Princeton, NJ 08544, USA
[2]Department of Physics, Harvard University, Cambridge, MA 02138, USA
[3]Center for Computational Quantum Physics, Flatiron Institute, 162 Fifth Avenue, New York, NY 10010, USA
[4]Department of Electrical Engineering and Computer Science, Massachusetts Institute of Technology, Cambridge, MA 02139, USA
[5]John A. Paulson School of Engineering and Applied Sciences, Harvard University, Cambridge, MA 02138, USA
[6]Menten AI, Inc., San Francisco, CA 94111, USA
[7]James Franck Institute, University of Chicago, Chicago, IL 60637, USA
[8]Computational Science Division, Argonne National Laboratory, Lemont, IL 60439, USA

NOISE MODEL DERIVATION

As discussed in the Noise Model section of the main text, we model the noisy application of an $R_x(\pm\pi/2)$ gate as an instantaneous rotation, followed by decay and dephasing over time t_* equal to the gate duration. Employing IBM's publicly available T_1 and T_2 times, along with the gate duration t_*, we define the following Kraus operators for the amplitude damping and phase damping noise channels, respectively:

$$A_0 = \begin{bmatrix} 1 & 0 \\ 0 & \sqrt{1-\lambda_{\mathrm{A}}} \end{bmatrix} \quad A_1 = \begin{bmatrix} 0 & \sqrt{\lambda_{\mathrm{A}}} \\ 0 & 0 \end{bmatrix} \tag{1}$$

$$P_0 = \begin{bmatrix} 1 & 0 \\ 0 & \sqrt{1-\lambda_{\mathrm{P}}} \end{bmatrix} \quad P_1 = \begin{bmatrix} 0 & 0 \\ 0 & \sqrt{\lambda_{\mathrm{P}}} \end{bmatrix} \tag{2}$$

The variables λ_{A} and λ_{P} are defined in Eqs. (6–7) of the main text, and their physical significance is described in the Noise Model section of the main text. Since the amplitude damping and phase damping superoperators commute, we can unambiguously define a mapping N that encapsulates the net effect of the two noise channels by applying one after the other, thereby mapping the density matrix

$$\rho = \begin{bmatrix} \rho_{00} & \rho_{01} \\ \rho_{10} & \rho_{11} \end{bmatrix} \tag{3}$$

to the density matrix

$$N(\rho) = \begin{bmatrix} \rho_{00}(1-\lambda_{\mathrm{A}}) + \lambda_{\mathrm{A}} & \rho_{01}\sqrt{1-\lambda_{\mathrm{A}}}\sqrt{1-\lambda_{\mathrm{P}}} \\ \rho_{10}\sqrt{1-\lambda_{\mathrm{A}}}\sqrt{1-\lambda_{\mathrm{P}}} & \rho_{11}(1-\lambda_{\mathrm{A}}) \end{bmatrix} \tag{4}$$

Using this mapping and the decomposition defined by Eq. (2) of the main text, we proceed by explicitly calculating the density matrix of a qubit after the noisy application of a single-qubit gate with Euler angles (β, γ, δ). If needed, we could begin with an initially mixed state parameterized by a 3-dimensional Bloch vector with length no more than unity, but for the sake of simplicity, we assume the input qubit is in an initially pure state with Bloch sphere coordinates (θ, ϕ). The initial state vector is given by the following:

$$|\psi(\theta,\phi)\rangle = \cos(\theta/2)\,|0\rangle + e^{i\phi}\sin(\theta/2)\,|1\rangle \tag{5}$$

The qubit's density matrix after the first pulse is given by:

$$\rho_1 = N(R_x(\pi/2)R_z(\delta)\,|\psi(\theta,\phi)\rangle\,\langle\psi(\theta,\phi)|\,R_z(\delta)^\dagger R_x(\pi/2)^\dagger) \tag{6}$$

After the second pulse:

$$\rho_2 = N(R_x(-\pi/2)R_z(\gamma)\rho_1 R_z(\gamma)^\dagger R_x(-\pi/2)^\dagger) \tag{7}$$

After the final $R_z(\beta)$ rotation:

$$\rho_{(\beta,\gamma,\delta,\theta,\phi)} = R_z(\beta)\rho_2 R_z(\beta)^\dagger \tag{8}$$

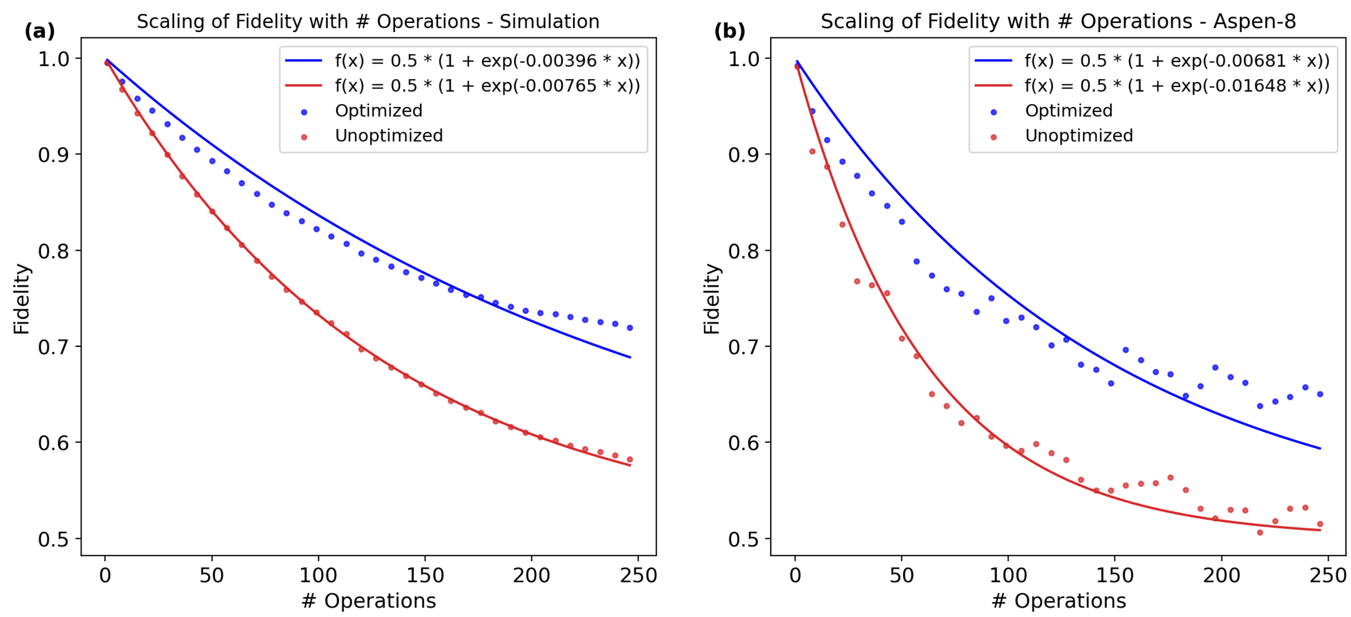

Figure S1: Scaling of fidelity with number of operations: the fidelity (vertical axis) representing the overlap between the state output by the noisy application of each circuit (unoptimized and optimized) and the target state output by the noiseless application of the unoptimized circuit is plotted against the circuit depth (horizontal axis) at which the fidelity was measured. Each data point is the average fidelity of 10 randomized gate sequences with 4,096 shots per measurement. Measurements were taken at circuit depths increasing by 7, $d \in \{1, 8, 15, ..., 246\}$.

Applying these transformations, we find that the noisy application of a single-qubit gate parameterized by Euler angles (β, γ, δ) transforms an initially pure state with Bloch sphere coordinates (θ, ϕ) into a mixed state with the density matrix defined in Eqs. (3–5) of the main text.

Throughout this derivation, we have implicitly assumed that the calibrated pulse amplitudes used to implement the $R_x(\pm\pi/2)$ gates in the presence of noise were exactly equal to the pulse amplitudes that would implement the $R_x(\pm\pi/2)$ gates perfectly in the absence of noise. This assumption is consistent with IBM's calibration methodology. IBM calibrates their $R_x(\pm\pi/2)$ gates by performing a Rabi experiment to determine the pulse amplitudes that most accurately implement them in the presence of noise [1–3]. Concretely, the optimal pulse is that which most accurately maps the $|0\rangle$ state to the state $R_x(\pm\pi/2)\,|0\rangle$ in the presence of noise. Within the framework of our noise model, since the angle of an R_x rotation is proportional to the amplitude of the pulse that implements it, finding the optimal pulse amplitude amounts to finding the optimal angle α that maximizes the following fidelity:

$$\begin{aligned} F &= \langle 0|\, R_x(\pm\pi/2)^\dagger N(R_x(\alpha)\,|0\rangle\,\langle 0|\, R_x(\alpha)^\dagger) R_x(\pm\pi/2)\,|0\rangle \\ &= \frac{1}{2}(1 \pm \sqrt{1-\lambda_{\mathrm{A}}}\sqrt{1-\lambda_{\mathrm{P}}}\sin\alpha) \end{aligned} \tag{9}$$

Since this achieves a maximum at $\alpha = \pm\pi/2$, it follows that the calibrated pulse amplitudes used to implement the $R_x(\pm\pi/2)$ gates in the presence of noise are exactly equal to the pulse amplitudes that would implement the $R_x(\pm\pi/2)$ gates perfectly in the absence of noise.

ERROR RATE REDUCTION ON ASPEN-8

Since Rigetti's QCs are built from superconducting transmon qubits, their native gate set is identical to IBM's, allowing for easy adaptation of our protocol. On Rigetti's Aspen-8 qubit 4 [4], we performed the same experiment outlined in the "Error Rate Reduction on `ibmq_rome`" subsection of the main text. Readout error was mitigated for all measurements made on Aspen-8 by inverting a calibration matrix [5] composed of Rigetti's publicly reported readout error probabilities. All single-qubit noise specifications for Aspen-8 were provided by Rigetti through PyQuil [4, 6, 7] and are tabulated in the Noise Specifications section below. The results of the experiment are displayed in

Fig. S1. An analysis of this data would mirror the analysis in the main text, so to avoid redundancy, we only discuss the reduction in error rate here.

On Aspen-8 qubit 4, our optimization protocol reduces the error rate of a single-qubit gate acting on a known initial state by 59%, from 8.2×10^{-3} to 3.4×10^{-3}. The unoptimized error rate that we report here does not agree with Rigetti's reported error rate of 7.5×10^{-3}, but there is better agreement than was found on `ibmq_rome`.

NOISE SPECIFICATIONS

For all of the QCs used throughout this work, the single-qubit noise specifications on their respective dates of use are tabulated below.

* Electronic address: maldonado@princeton.edu

[1] S. Ashhab, J. Johansson, and F. Nori, New Journal of Physics **8**, 103 (2006).
[2] Y. M. Galperin, D. V. Shantsev, J. Bergli, and B. L. Altshuler, Europhysics Letters (EPL) **71**, 21 (2005).
[3] J. Majer, J. M. Chow, J. M. Gambetta, J. Koch, B. R. Johnson, J. A. Schreier, L. Frunzio, D. I. Schuster, A. A. Houck, A. Wallraff, and et al., Nature **449**, 443447 (2007).
[4] Rigetti Computing Quantum Cloud Services, http://qcs.rigetti.com, accessed August, 2020.
[5] B. Nachman, M. Urbanek, W. A. de Jong, and C. W. Bauer, npj Quantum Information **6**, 1 (2020).
[6] R. S. Smith, M. J. Curtis, and W. J. Zeng, arXiv preprint arXiv:1608.03355 (2016).
[7] P. J. Karalekas, N. A. Tezak, E. C. Peterson, C. A. Ryan, M. P. da Silva, and R. S. Smith, Quantum Science and Technology **5**, 024003 (2020).

Noise Specifications - ibmq_rome - 07/14/20						
Qubit	T_1 [μs]	T_2 [μs]	Pulse Duration [ns]	Prob. Prep. $\|0\rangle$ Meas. $\|1\rangle$	Prob. Prep. $\|1\rangle$ Meas. $\|0\rangle$	Gate Error [10^{-4}]
0	116	93.4	35.6	0.030	0.063	3.23
1	105	55.2	35.6	0.020	0.073	3.00
2	80.2	124	35.6	0.073	0.087	4.04
3	46.4	105	35.6	0.027	0.050	3.35
4	71.6	138	35.6	0.027	0.043	5.01
Mean	83.9	103	35.6	0.035	0.063	3.77
SD	27.7	31.7	0.00	0.022	0.017	0.798

Noise Specifications - ibmq_bogota - 08/10/20						
Qubit	T_1 [μs]	T_2 [μs]	Pulse Duration [ns]	Prob. Prep. $\|0\rangle$ Meas. $\|1\rangle$	Prob. Prep. $\|1\rangle$ Meas. $\|0\rangle$	Gate Error [10^{-4}]
0	126	158	35.6	0.024	0.108	5.10
1	117	168	35.6	0.004	0.039	2.36
2	107	142	35.6	0.009	0.026	2.19
3	199	240	35.6	0.005	0.034	3.96
4	142	249	35.6	0.019	0.135	2.02
Mean	138	192	35.6	0.012	0.068	2.63
SD	36.4	49.6	0.00	0.009	0.050	0.898

Noise Specifications - Aspen-8 - 08/30/20						
Qubit	T_1 [μs]	T_2 [μs]	Pulse Duration [ns]	Prob. Prep. $\|0\rangle$ Meas. $\|1\rangle$	Prob. Prep. $\|1\rangle$ Meas. $\|0\rangle$	Gate Error [10^{-4}]
0	16.9	18.7	60.0	0.014	0.034	50.0
1	36.7	40.4	60.0	0.049	0.063	4.00
2	18.5	12.1	60.0	0.017	0.033	112
3	34.2	19.5	60.0	0.022	0.046	13.0
4	15.3	17.6	60.0	0.017	0.034	75.0
5	41.4	5.37	60.0	0.047	0.075	5.00
6	45.0	45.1	60.0	0.015	0.042	31.0
7	17.2	22.8	60.0	0.016	0.038	11.0
11	23.8	12.7	60.0	0.058	0.058	10.0
12	21.6	8.18	60.0	0.034	0.134	27.0
13	27.0	18.3	60.0	0.040	0.056	26.0
14	17.9	11.3	60.0	0.018	0.037	40.0
15	35.9	3.76	60.0	0.035	0.066	16.0
16	21.8	28.6	60.0	0.009	0.037	24.0
17	37.9	14.7	60.0	0.021	0.042	13.0
20	18.7	15.7	60.0	0.017	0.041	19.0
21	43.7	7.29	60.0	0.045	0.082	32.0
22	29.6	25.3	60.0	0.035	0.072	19.0
23	24.8	9.89	60.0	0.074	0.078	1034
24	12.9	2.18	60.0	0.042	0.116	18.0
25	42.9	20.4	60.0	0.035	0.060	10.0
26	10.6	2.22	60.0	0.015	0.047	79.0
27	43.1	18.7	60.0	0.036	0.074	8.00
30	21.1	26.3	60.0	0.040	0.178	70.0
31	42.0	37.2	60.0	0.035	0.092	5.00
32	43.1	56.8	60.0	0.022	0.076	10.0
33	29.0	27.0	60.0	0.027	0.080	9.00
34	17.9	21.0	60.0	0.010	0.034	15.0
35	30.5	35.3	60.0	0.052	0.091	2.00
36	33.8	23.4	60.0	0.041	0.091	45.0
37	35.3	22.8	60.0	0.041	0.053	10.0
Mean	28.7	20.3	60.0	0.032	0.066	59.4
SD	10.7	12.8	0.00	0.016	0.033	183
